# Mitochondria Transfer from Adipose Stem Cells Improves the Developmental Potential of Cryopreserved Oocytes

**DOI:** 10.3390/biom12071008

**Published:** 2022-07-21

**Authors:** Udayanga Sanath Kankanam Gamage, Shu Hashimoto, Yuki Miyamoto, Tatsuya Nakano, Masaya Yamanaka, Akiko Koike, Manabu Satoh, Yoshiharu Morimoto

**Affiliations:** 1 HORAC Grand Front Osaka Clinic, Osaka 530-0011, Japan; miyamoto313@ivfjapan.com (Y.M.); koike027@ivfjapan.com (A.K.); 2 Reproductive Science Institute, Graduate School of Medicine, Osaka Metropolitan University, Osaka 545-8585, Japan; 3 IVF Namba Clinic, Osaka 550-0015, Japan; nakano@ivfnamba.com (T.N.); yamanaka@ivfnamba.com (M.Y.); satou@ivfnamba.com (M.S.)

**Keywords:** oocyte cryopreservation, mitochondria supplementation, embryo development, adipose stem cell

## Abstract

Although it is not a well-established technology, oocyte cryopreservation is becoming prevalent in assisted reproductive technologies in response to the growing demands of patients’ sociological and pathological conditions. Oocyte cryopreservation can adversely affect the developmental potential of oocytes by causing an increase in intracellular oxidative stresses and damage to the mitochondrial structure. In this study, we studied whether autologous adipose stem cell (ASC) mitochondria supplementation with vitrified and warmed oocytes could restore post-fertilization development that decreased due to mitochondrial damage following cryopreservation. ASC mitochondria showed similar morphology to oocytes’ mitochondria and had a higher ATP production capacity. The vitrified-warmed oocytes from juvenile mice were supplemented with ASC mitochondria at the same time as intracellular sperm injection (ICSI), after which we compared their developmental capacity and the mitochondria quality of 2-cell embryos. We found that, compared to their counterpart, mitochondria supplementation significantly improved development from 2-cell embryos to blastocysts (56.8% vs. 38.2%) and ATP production in 2-cell embryos (905.6 & 561.1 pmol), while reactive oxygen species levels were comparable. With these results, we propose that ASC mitochondria supplementation could restore the quality of cryopreserved oocytes and enhance the embryo developmental capacity, signifying another possible approach for mitochondrial transplantation therapy.

## 1. Introduction

In assisted reproductive therapies, the cryopreservation of oocytes has been widely occupied in helping meet the inevitable sociological and pathological requirements. However, the developmental efficacy of cryopreserved oocytes is lower than that of fresh oocytes [1,2]. It is well known that during cryopreservation, the osmolarity of the extracellular environment is increased by non-permeant cryoprotectants like sucrose to promote a water efflux from the cells, whereas the permeant cryoprotectant substitute intracellular water decreasing the freezing point of cytoplasm. It has been reported that unfertilized oocytes are more vulnerable to cryopreservation than zygotes, as a phase transition of membrane lipids occurs at fertilization [3]. Furthermore, unfavorable reduction of mitochondria membrane potential (MMP) and damage to mitochondrial structures have been reported as having detrimental effects on oocytes following cryopreservation [4,5]. Cryopreservation-mediated oxidative stress could also adversely affect oocyte quality and post-fertilization development [5,6,7,8], and may be correlated with increased embryonic fragmentation and slow cleavage [6,7,9,10,11,12]. Thus, the post-implantation developmental potential of embryos derived from vitrified oocytes is still remarkably lower than fresh oocytes [1,13], necessitating further exploration and improvement of the cryopreservation technique.

In the mitochondria of fresh oocytes, the MMP varies according to the cellular localization of mitochondria. Primarily, mitochondria are localized in the different regions of the cytoplasm according to the MMP, and this localization pattern may be important to regulate successful embryo development [14]. It is reported that this functional mitochondria localization can be seriously damaged by oocyte cryopreservation, adversely affecting post-fertilization development [15]. Additionally, ultra-structural mitochondrial damages have been reported following oocyte cryopreservation, which could reduce early embryos’ development potential while increasing apoptosis [16,17]. Further, mitochondrial damage in slow-cooled human oocytes has been shown to delay the recovery of intracellular Ca^2+^ basal levels after artificial oocyte activation through ionophore. This suggests that cryopreservation could damage the development of calcium transients at fertilization [18]. There have been many pre-clinical level research studies that mainly sought to improve mitochondrial biogenesis [19], decrease ROS production [20], and reestablish Ca^2+^ ion regulation [21] in cryopreserved oocytes. Although these pre-clinical studies showed some improvement in embryo development, none have been able to restore the damaged oocyte mitochondria following cryopreservation. It is therefore essential to have a high and optimum number of intact mitochondria in the mature oocyte, as they have been designed to distribute mitochondria to the early and post-implantation embryo cells where mitochondria replication is absent [22]. Moreover, the availability of active mitochondria in the 1- to 2-cell stage embryos is vital for developing early preimplantation embryos. And defected mitochondria in mature oocytes could arrest the embryo’s development at the 2-cell stage [23]. Thus, mitochondria supplementation to vitrified-warmed mature oocytes in an autologous manner may efficiently restore weakened biogenesis from mitochondrial damages following the oocyte cryopreservation.

There have been several attempts to improve the advanced aged oocyte quality by manipulating mitochondrial functions and the number of mitochondria in the oocytes [24,25]. Ooplasm transfer, GV/pronuclear transfer, and autologous stem cell mitochondria transplantation are the main approaches to increasing the number of mitochondria copies in oocytes. However, due to the risk of heteroplasmy, ooplasm transfer and GV/pronuclear transfer are no longer available in clinical practices [26]. The supplementation of mitochondria from an autologous oogonial stem cell has recently been tested in different clinical groups targeting rejuvenation of the quality of advanced-age oocytes [24,27,28]. However, due to counterarguments related to the experimental approaches and the cell specificity, the positive outcome results were not feasible enough to continue with human subjects.

It was recently confirmed that autologous adipose stem cell (ASC) and induced pluripotent stem cell mitochondria could improve the quality of aged mouse oocytes [29,30]. Furthermore, mitochondria isolated from liver cells or follicular granulosa cells may play different roles in oocytes due to diverse mitochondrial structures and functions of different cell types [31,32]. However, there is no previous study that has confirmed the influence of adipose stem cell mitochondria supplementation on the developmental potential of cryopreserved oocytes. This study analyzes whether ASC mitochondria supplementation during the ICSI process can revitalize the compromised quality of cryopreserved oocytes. From these findings, for the first time, we propose that autologous ASC mitochondria supplementation may be a promising strategy to improve developmental potential through the compensation of cryopreservation-related mitochondrial damages to oocytes.

## 2. Materials and Methods

### 2.1. Animals

All experiments were performed following the guidelines of the Institutional Animal Care Ethics Committee and Use Committee of Osaka City University in Osaka, Japan. (Experiment approval no: 21061). Female and male C57BL/6JJmsSlc and ICR mice (4–8 weeks) were purchased from SLC Japan. The mice were housed in a room maintained under regular controlled SPF environmental conditions, with a temperature of 22–24 °C and a 12-h light/dark cycle.

### 2.2. Ovarian Super-Stimulation, Oocyte Collection

Young female C57BL/6JJmsSlc mice (8 weeks) were superovulated through an IP injection of 5 IU of pregnant mare serum gonadotropin (PMSG) (Serotropin ASKA Animal Health Co., Ltd., Tokyo, Japan) followed by an IP injection 48 h later with 5 IU of human chorionic gonadotropin (hCG). Unfertilized oocytes were collected from the oviducts at 14–15 h post hCG injection, and cumulus cells were denuded from cumulus-oocyte complexes using 0.3 mg/ml of hyaluronidase at 37 °C for less than 5 min. The remaining cumulus cells were removed by repeated aspiration with a narrow pipette. Cumulus-free oocytes were washed three times in an M2 medium (Sigma-Aldrich, St. Louis, MO, USA). The mature oocytes were cultured in a humid incubator at 37.0 °C under 5% CO_2_ for approximately 30 min until vitrification started.

### 2.3. Vitrification of Oocytes

The vitrification and warming protocol of this experiment was based on the Cryotop kit instructions (Kitazato BioPharma, Shizuoka, Japan) using equilibration solution (ES: 7.5% (*v*/*v*) ethylene glycol (EG) and 7.5% (*v*/*v*) dimethyl sulfoxide (DMSO) and vitrification solution (15% (*v*/*v*) EG, 15% (*v*/*v*) DMSO) and performed according to a previous study [32]. The oocytes were briefly washed in modified human tubal fluid (mHTF; Irvine Scientific, Santa Ana, CA, USA); then, they were equilibrated in 50% (*v*/*v*) ES solution for 3 min, then in 67% (*v*/*v*) ES solution for 3 min, and finally in ES solution for 7 min at room temperature. Subsequently, the oocytes were transferred to the vitrification solution for 30 s. After 25–30 s, 5–10 oocytes were placed on the Cryotop with a small amount of vitrification solution (approximately 0.4 μL). The excess amount of vitrification solution (the vitrification solution not covering the oocytes) was aspirated, and the Cryotop containing the oocytes was plunged into liquid nitrogen (LN2).

### 2.4. Warming of Oocytes

On the day of ICSI, the vitrified oocytes were warmed by transferring them to a pre-warmed thawing solution at 37 °C. After 1 min, the oocytes were transferred to a diluent solution for 3 min at 37 °C. Subsequently, the oocytes were transferred to a washing solution for 5 min at 37 °C. Oocytes were then washed in mHTF and incubated for at least 30 min at 37 °C under 5% CO_2_ before ICSI.

### 2.5. Cell Isolation, Culture, and Confirmation

Following a published protocol with slight modifications, adipose-derived stem cells were isolated from mouse inguinal adipose tissues [33]. Briefly, both sides of inguinal subcutaneous fat tissues were aseptically collected from female C57BL/6JJmsSlc mice selected for oocyte retrievals. The adipose tissues were either cryopreserved by adding 9% glycerol, 0.2 M sucrose cryopreservation medium (Cooper Surgical Company, Trumbull, CT, USA) or directly minced and digested in 0.1% (*w*/*v*) collagenase type 1 (Roche Diagnostics, Basel, Switzerland). Then, a cell suspension was prepared by mechanical and enzymatic dissociation of the tissue with gentleMACS™ Octo dissociator (Miltenyi Biotech, Bergisch Gladbach, Germany) at 37 °C for 40 min. This suspension was then centrifuged at 800× *g* for 10 min, and the supernatant was discarded. The stromal vascular fraction was resuspended and washed twice in an R-STEM medium (Rohto Pharmaceutical Co., Ltd., Osaka, Japan). Then, the cell fraction was filtered with a 70-μm cell strainer (Corning incorporated, Corning, NY, USA) and centrifuged at 500× *g* for 5 min to obtain a high-density cell pellet. The cells were then cryopreserved by using 9% glycerol, 0.2 M sucrose cryopreservation medium or cultured in an R-STEM medium with 5% fetal bovine serum in a humidified environment with 5% CO_2_ (*v*/*v*) at 37 °C. Unattached and residual non-adherent red blood cells were removed by washing them with PBS 24–48 h later. The culture medium was changed every 48 h until the cells reached 90% confluency. The cells were passaged at a density of 1 × 10^4^ cells/cm^2^, or the cells at passage three were cryopreserved for mitochondria isolation with a 9% glycerol, 0.2 M sucrose cryopreservation medium.

### 2.6. Analysis of the Specificity of ASC

After collecting single-cell fractions from the adipose tissues, the cells were incubated in 2% human serum albumin for 20 min at RT. The cells were then incubated with Alexa fluor 647 anti-mouse CD29 (Biolegend, Tokyo, Japan), Alexa fluor 488 anti-mouse CD105 (Biolegend), phycoerythrin anti-mouse CD90 (Novus Biologicals, Littleton, CO, USA), FITC anti-mouse CD45 (Invitrogen, Waltham, MA, USA) fluorescent conjugated antibodies or isotype control Alexa flour 647 anti-mouse IgG1(Biolegend), and FITC anti-mouse IgG1 (Biolegend) antibodies according to recommended concentration and incubated for 1 h at RT. After that, the cells were washed, and fluorescence-activated cell sorting (FACS; Sony, Tokyo, Japan) was used to analyze the availability of ASC in the live cell fraction, confirming the positive expression of CD29(+) and CD105(+), and negative expression of CD45(−) compared to the isotype control.

The cell specificity of cultured cells was analyzed for specific cell surface antigens with flow cytometry and FlowJo software (FlowJo, version 7.6.1, BD Life Sciences, Ashland, OR, USA). Briefly, single-cell suspensions were harvested using 0.05% trypsin/EDTA (Sigma-Aldrich, St. Louis, MO, USA) and resuspended in Hanks’ Balanced Salt Solution (HBSS, Thermo Fisher Scientific, Waltham, MA, USA). The cells were incubated for 1 h RT with CD105, CD90, CD29, or CD45 or isotype control antibodies. After thorough washing, the cells’ surface expressions were analyzed.

For microscopic fluorescence analysis, cultured cells were fixed with 4% paraformaldehyde for 2 h at RT and washed three times in HBSS. Then the cells were incubated with 2% fetal bovine serum for 20 min and incubated with the above CD29 and CD105 antibodies for 1 h. After that, the cells were washed with HBSS, and 4′,6-Diamidino-2-phenylindole Dihydrochloride (DAPI, Dojindo, Kumamoto, Japan) was added before analysis with fluorescence microscopy (Keyence, Osaka, Japan).

Adipogenic and osteogenic differentiation assays assessed the multipotency of ASCs. In brief, adipogenesis was induced by an adipogenic induction medium (PromoCell, Heidelberg, Germany) for 14 days and confirmed by light microscopy as an indicator of intracellular lipid accumulation. Osteogenesis was induced by culturing ASCs in the osteogenic induction medium (PromoCell) on 1% fibronectin-coated (Sigma-Aldrich, St. Louis, MO, USA) 24-well plates for 28 days. Calcium deposition was analyzed with alizarin red staining.

### 2.7. Mitochondria Membrane Potential Analysis

The MMP was detected by the JC-10 MMP Assay Kit (AAT Bioquest, Inc., Sunnyvale, CA, USA). Oocytes, embryos, and cells were stained with 10 μg/mL JC-10 dye for 30 min at 37 °C under 5% CO_2_. The distribution of JC-10 monomers (green fluorescence) and JC-10 aggregate fluorescence (red fluorescence) was determined by fluorescence microscopy (Keyence) or FACS (Sony, Tokyo, Japan). The captured images or expression intensities were processed using Image J or FlowJo software. The mitochondrial membrane potentials were assessed by measuring the ratios of red to green fluorescence.

### 2.8. ATP Production Analysis

ATP level was measured according to the manufacturers’ instructions using Luciferase Assay Kit-Firefly (Abcam, Cambridge, UK). The ASC, adipose cell, oogonial stem cell, splenic cell, and hepatic cell were isolated from three female animals and cultured at 37 °C overnight. The mouse ovarian stem cells were isolated according to a previously reported protocol [33]. In brief, a cell suspension was prepared by mechanical and enzymatic dissociation of ovaries using gentleMACS™ Dissociator (Miltenyi Biotech, Bergisch Gladbach, Germany). The isolated cells were stained with fluorescein-labeled monoclonal or polyclonal anti-DDX4 (VASA) antibodies (Bioss Antibodies, Beijing, China), and OSCs were isolated using fluorescence-activated cell sorting (FACS) on a Sony SH800 cell sorter (Sony Biotechnology Inc., San Jose, CA, USA). (Supplementary Figure S1B). The same number of each cell type was used (2000 cells per group) for ATP level analysis. All the cells were cryopreserved in 10 μL of Milli-Q water and warmed before adding the luciferase assay reagent, which damages the cell wall and allows ATP to escape and be exposed to luciferase. At the time of analysis, the cells were moved into a transparent tube in 50 μL of Milli-Q water and incubated for 10 min after adding 50 μL of luciferase at RT. In the presence of ATP, luciferase catalyzes the bioluminescent oxidation of luciferin. The intensity of the light source was analyzed with a luminometer (Microtec, Chiba, Japan). To analyze the ATP level in 2-cell embryos, randomly selected individual 2-cell embryos from three consecutive ICSI (*n* = 14, mito-ICSI & cont-ICSI) were used following the above protocol.

### 2.9. ROS Analysis

2-cell embryos were randomly selected and washed in Ca^2+^ and Mg^2+^ free PBS (PBS (−) supplemented with 0.1% polyvinyl alcohol) (PVA-PBS) and stained with 1 μM H2DCFDA (D399, Thermo Fisher Scientific, Waltham, MA,, USA) PVA-PBS for 10 min at room temperature (RT) under light protection as previously reported [34]. At the start of each experiment, fresh H2DCFDA was prepared at 1 μM in PVA-PBS from stock solution (1 mM in DMSO). Stained 2-cell embryos were washed in PVA-PBS to remove the residuals of the dye and were transferred into a 3 μL droplet of PVA-HTF on a glass-bottomed culture dish (P35G-0-14-C; MatTek Corporation, Ashland, MA, USA). Microscopic images were obtained using a CLM (CellVoyager CV1000; Yokogawa Electronics, Tokyo, Japan) at 40× at RT in air, and fluorescence intensity in the equatorial plane of oocytes was measured using ImageJ (http://imagej.nih.gov/ij/) (accessed on 21 January 2022).

### 2.10. Transmission Electron Microscopy Analysis

ASCs or oocytes were fixed in cold 2% glutaraldehyde in cacodylate buffer for 1 h at 4 °C, followed by post-fixation in 1% aqueous osmium tetroxide for 1 h. They were rapidly dehydrated in graded ethanol solutions with a final rinse in acetone and embedded in Epon. Semithin sections were stained with toluidine blue. Ultrathin sections were stained with uranyl acetate and lead citrate before examination under a transmission electron microscope (JEM1011, JEOL, Tokyo, Japan).

### 2.11. Mitochondria Isolation and Supplementation

**Figure 1:**
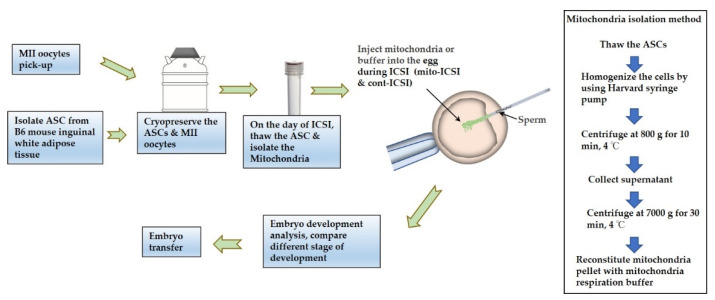
Schematic workflow of the study including the isolation of ASC, isolation of mitochondria, supplementation to oocytes, and analysis of effectiveness.

To attain the minimum required number of mitochondria (500 mtDNA copy) to improve ICSI results, the isolation of mitochondria from ASCs was performed following previously described protocols with minor modifications [35,36]. In brief, 2.5 × 10^5^ cells were used for mitochondria isolation throughout all the experiments. The cells were washed in HBSS and then resuspended in an extraction buffer (420 mM D mannitol, 140 mM sucrose, 10 mM HEPES, 2 mM KCl and 2 mM EGTA, pH 7.2) with 0.5% human serum albumin (Irvine Scientific, Santa Ana, CA, USA). The cells were then homogenized for 10 min at 5 °C by repeated infusion and withdrawal using a syringe pump connected to a 25 g cannula (Harvard Apparatus, Holliston, MA, USA). The homogenates were centrifuged at 800× *g* for 10 min before the supernatant was carefully collected. The supernatant was then centrifuged at 7000× *g* for 30 min at 5 °C to separate mitochondria from smaller cell debris. After the supernatant was discarded, mitochondrial pellets were kept in a respiration buffer (225 mM D mannitol, 200 mM sucrose, 10 mM KCl, 10 mM Tris-HCl, and 5 mM KH2PO4, pH 7.2) with 0.5% human serum albumin until use. Approximately 2–3 pL of mitochondrial suspension and a spermatozoon were injected together (mito-ICSI) into an oocyte, and within 3 h mitochondria isolation was completed. The control group was injected only with respiration buffer (cont-ICSI) (Figure 1).

### 2.12. Embryo Development Analysis

The oocyte warming and mitochondria isolation process was done immediately before the ICSI process began. Following the mitochondria injection and cont-ICSI completion, the injected oocytes were incubated in KSOMaa (Ark Resource, Kumamoto, Japan) under mineral oil at 37 °C, 5% CO_2_, and 5% O_2_. First, the survival rate was analyzed according to the total injected oocytes. After approximately 4 h, the fertilization rate (and on day 1 the 2-cell embryo rate and on day 4 [after 96 h]), the blastulation number and the rate were analyzed.

### 2.13. Statistical Analysis

All data are presented as mean ± SEM. Unpaired Student t-test was used to compare differences among groups using Graphpad prism software (Ver. 5, GraphPad Inc., San Diego, CA, USA). *p* < 0.05 was considered statistically significant.

## 3. Results

### 3.1. Mouse Adipose Stem cell (ASC) Isolation and Confirmation

**Figure 2:**
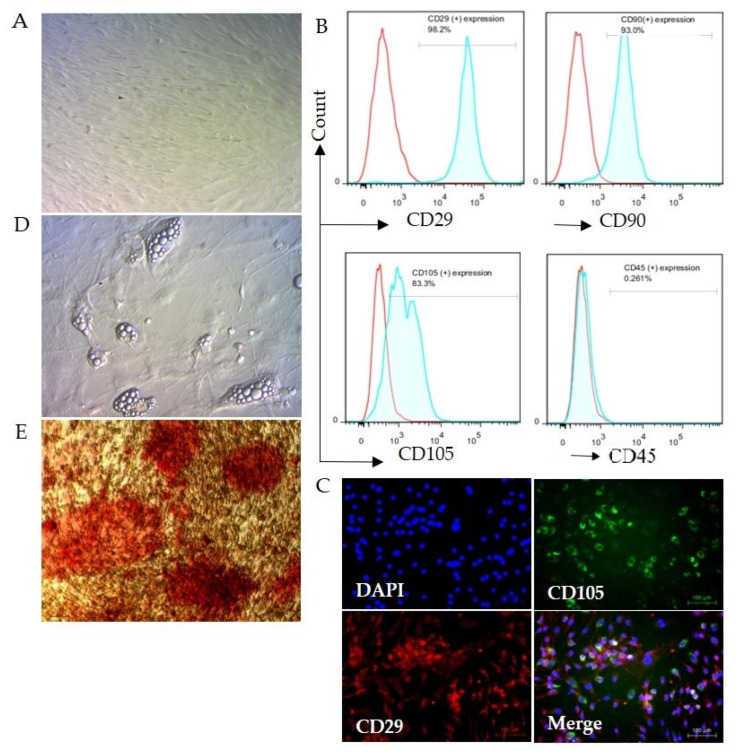
Mouse adipose stem cell (ASC) isolation and confirmation. (**A**). Morphology of ASC shows regular fibroblastic cell morphology. (**B**). Flow cytometric analysis of cultured ASC at 3rd passage. ASC was positive for CD29, CD90, and CD105 and negative for CD45, which confirms the purity of ASC. (**C**). Immunofluorescence staining confirmed the expression of CD29 and CD105 in ASCs. The nucleus was stained with DAPI. (**D**,**E**). Confirmation of the multipotency of ASC. (**D**). Adipogenic differentiation. Oblate-like cell morphology and numerous lipid droplets can be visualized without specific staining. E. Osteogenic differentiation. The cells developed a cluster-shaped structure and showed positive red staining with alizarin red staining, confirming the multipotency of ASC.

Adipose cells were isolated from female C57BL/6JJmsSlc mice’s inguinal subcutaneous adipose tissues. The isolated cells were analyzed to confirm ASC specificity through fluorescent activated cell sorting (FACS) after three passages of the culture. Confirming the specificity of ASC, the cells showed positive expression of anti-CD29, anti-CD90 & anti-CD105 antibodies, and negative expression of CD45, as previously reported [37,38] (Figure 2A).

Following the culture of isolated cells, they resembled spindle/fibroblast shapes on day 3, and symmetric colonies were also observed (Figure 2B). To confirm the specificity of ASC, the positive expressions of CD29 and CD105 antibodies were confirmed with fluorescent microscopy (Figure 2C). Moreover, in confirming the multipotency of the ASC used in this study, we differentiated between adipocytes and osteoblasts. After the culture was in an adipogenic differentiation medium for 7–10 days, adipogenicity could be confirmed even without using Oil Red O staining, as the morphology of the ASCs changed from spindle-like to oblate-like, and numerous lipid droplets appeared in the cells/dishes (Figure 2D). After culture in an osteogenic medium for 28 days, morphological changes in culture cells were shown as they developed cluster-shaped structure and were positive on alizarin red staining (Figure 2E), demonstrating multipotency phenotype, which is a basic specific feature of adipose-derived stem cells [37].

### 3.2. Mouse Adipose Stem Cell Mitochondria Have High Membrane Potentials and ATP Production Capacity

**Figure 3:**
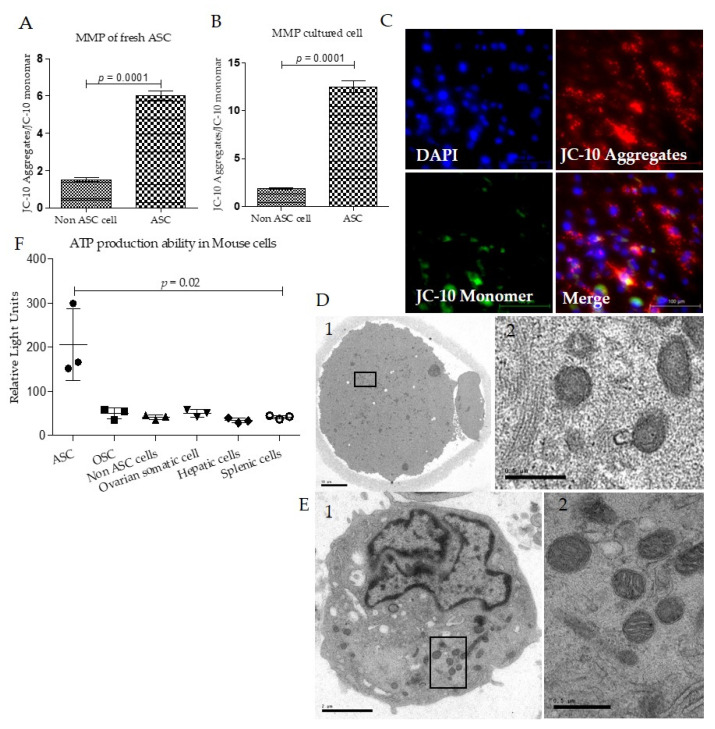
Mitochondrial characteristics of mouse adipose stem cells (ASC)and mature oocytes. (**A**). The ASC and non-ASC were sorted from the single-cell fraction isolated from adipose tissues. Additionally, mitochondrial membrane potential (MMP) was analyzed through staining with JC-10 and using FACS. (**B**). The cultured ASC (at passage 3) was analyzed for MMP, and both fresh and cultured ASCs show higher MMP than non-ASC cells. (**C**). The representative fluorescence micrographs show the JC-10-stained cultured ASC (**D**). Representative transmission electron micrograph (TEM) of mature mouse oocyte (scale bar—10 µm) and higher magnification of oocyte mitochondria (scale bar—0.5 µm). (**E**). Representative transmission electron micrograph of ASC (scale bar—2 µm) higher magnification of ASC mitochondria (scale bar—0.5 µm) show spherical shape mitochondria. (**F**). The ATP production ability (RLU, relative light unit) is significantly higher in ASC compared with different cell types such as ovarian stem cell (OSC), non-ASC cells, ovarian somatic cells, hepatic cells, and splenic cells. Data in the graphs are represented as mean ± SD.

JC-10 staining was used to analyze the mitochondrial membrane potentials in ASC. The membrane potentials were analyzed according to the ratio of median fluorescence intensity of JC-10 aggregates (red) and JC-10 Monomer (green). Both freshly isolated ASC and cultured ASC showed higher membrane potentials than non-ASC cells, as reported in the previous study [39]; (Figure 3A,B).

Most ASCs showed higher aggregation of JC-10 in mitochondria, demonstrating higher mitochondria membrane potentials (Figure 3C). To identify the morphology of the mitochondria, we analyzed the ultrastructure of the mature mouse oocyte (Figure 3D) and ASC (Figure 3E) with transmission electron microscopy. Mature oocytes comprise round and spherical shape mitochondria (Figure 3D2). The ASC contained a comparatively small nucleus and a higher number of mitochondria. Additionally, most of the mitochondria were round and spherical and had a deficient number of cristae, resembling oocyte mitochondria (Figure 3E2). Finally, we analyzed the ATP production capacity of ASC. We compared it with various other mouse cell types, non-ASC cells, oogonial stem cells, ovarian somatic cells, hepatic cells, and splenic cells, after which ASC clearly showed higher ATP production capacity than other cell types (Figure 3F). All these data confirmed that ASC had active mitochondria that morphologically resembled mitochondria of oocytes.

### 3.3. Adipose Stem Cell Mitochondria Supplementation during the ICSI Process Improves Embryo Development and Live Birth Capacity

**Figure 4:**
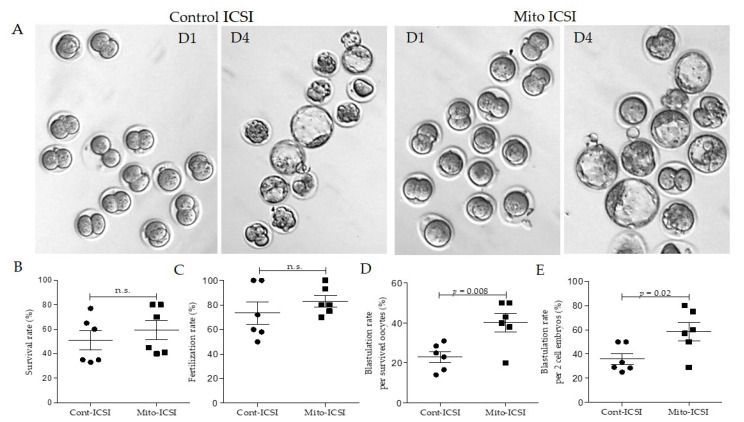
Adipose stem cell mitochondria injection improves blastulation development potentials. (**A**). Representative micrograph of day 1 and day 4 stages of embryos in control and mitochondria-supplemented groups. (**B**,**C**) The survival rates and fertilization rates were comparable in both groups after ICSI. (**D**,**E**). A significantly higher blastulation rate was present in the mitochondria supplemented group compared to the control group, (**D**) per survived oocytes and (**E**) per 2-cell embryos.

In humans and mice, vitrified-warmed oocytes show lower developmental potentials than fresh oocytes [40,41,42]. In this study, we have used vitrified oocytes after warming for all the ICSI procedures. Therefore, the average fertilization rate, two-cell embryo development rate, and blastulation rate are generally lower than ICSI performed with fresh oocytes. According to a previous study, the vitrified-warmed oocytes of C57BL/6 mice show around 46% of 2-cell development capacity and around 23% blastulation rate from cleaved embryos following the ICSI [40]. In our study, approximately the same number of oocytes were used for the mito-supplemented and cont-ICSI groups (mito-ICSI & cont-ICSI). The average values for different development stages are shown in Table 1. The survival rates after ICSI were 50.4% and 58.6%, the fertilization rates were 75.4% and 86.8%, and the development rates up to the 2-cell stage were 52.9% and 60.9% for cont-ICSI and mito-ICSI, respectively. These values didn’t significantly differ (Table 1, Figure 4A–C).

However, mitochondria supplementation significantly improved the blastulation rate of surviving oocytes (*p* < 0.05, 41% vs. 25%) and 2-cell embryos (*p* < 0.05, 56.8% vs. 38.6%) compared to the cont-ICSI group (Table 1. Figure 4A,D,E).

Table 1 Detailed developmental data of control and mito-ICSI of cryopreserved oocytes. Approximately the same number of oocytes was used in both groups. Though it is not statistically significant, the survival rate, fertilization rate, and 2-cell embryo development rates were slightly improved following the mitochondria supplementation with ICSI. Blastulation rates per survived oocytes and 2-cell embryos were significantly higher in the mito-ICSI group compared to the cont-ICSI group. Data are shown as mean ± SD.

From these data, we’ve concluded that oxidative stress and other cellular stress-mediated mitochondrial damages arise during cryopreservation procedures and that supplementing autologous mitochondria with ICSI procedures can revitalize cryo-damaged oocytes.

### 3.4. The Mitochondria Supplemented Group Has Higher Active Mitochondria in Early Embryos

**Figure 5:**
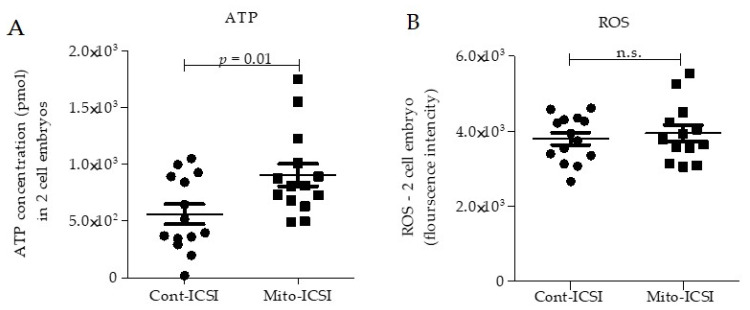
Mitochondria supplementation enhances the ATP production capacity in 2cell embryos. (**A**). ATP level in 2-cell embryos in the mitochondria supplemented group was significantly higher compared to the control group. (**B**). The ROS level was comparable in both groups. Data in the graphs were represented as mean ± SD.

To identify why ASC mitochondria supplementation may improve the developmental potential of preimplantation embryos, we analyzed ATP content and ROS levels in 2-cell embryos. ATP level in two-cell embryos in mito-ICSI was significantly higher than in the cont-ICSI group (Figure 5A).

Furthermore, active mitochondria and the availability of higher mitochondria numbers in the early stage of embryos were important in developing early preimplantation embryos [23,43]. This finding illustrates that ASC mitochondria supplementation could restore overall mitochondrial function in early embryos damaged by oocyte cryopreservation. However, the ROS level is similar in 2-cell embryos of both the mito-ICSI and the cont-ICSI groups (Figure 5B). This may be due to the random selection of 2-cell embryos for ROS analysis and the fact that ROS levels can fluctuate widely in early embryos according to the cell division status [44].

## 4. Discussion

Oocyte cryopreservation has shown a dramatic upsurge in use over the last decade [45]. This is undoubtedly because it is one of the greatest advances in fertility preservation, providing the possibility of bearing children using one’s own gametes to women who face social obligations, age-related decline in fertility, or want to have children after receiving antineoplastic therapies for cancer [46]. Though the composition of cryoprotectants and the cryopreservation protocol have significantly advanced since the introduction of oocyte cryopreservation, the overall success rate is still far behind that of fresh oocytes [2]. Intracellular damages, including mitochondrial structural and functional impairments, are significant negative influences resulting from oocyte cryopreservation [5]. This study analyzed whether autologous ASC mitochondria supplementation could restore post-fertilization development in vitrified-warmed oocytes. Compared to the control group, the blastulation and quality of 2-cell embryos were significantly improved after mitochondria supplementation in cryopreserved juvenile mouse oocytes. Our findings, therefore, provide convincing evidence that mitochondria supplementation could substitute compromised mitochondrial functions in cryopreserved oocytes.

In cryopreserved oocytes, the distribution and dynamics of mitochondria are disturbed, and the membrane potential is lowered, resembling the condition of aged oocytes [47]. It has also been reported that human and animal oocytes’ mitochondrial structure and function are seriously diminished following cryopreservation [17,48,49,50]. The cryoprotectant may cause Ca^2+^ overload and increase ROS, causing the prolonged opening of mitochondrial permeability transition pore and triggering further ROS generation, rupture of mitochondrial membranes, Cyt C release, and eventually inducing apoptosis. The sub-lethal damage in the mitochondria of warmed oocytes can reduce their developmental capacity [5]. Further, owing to their large cytoplasm, mammalian oocytes could be vulnerable to structural and functional damages during the cryopreservation process [5]. However, the mtDNA copy number does not increase during early embryogenesis until the blastocyst stage [43,51,52,53]. During early embryogenesis, energy metabolism depends mainly on pyruvate (and fatty acids in some species) via oxidative phosphorylation [54,55]. And abnormal functioning of oocyte mitochondria may be eliminated by mitophagy or bottleneck phenomenon [56,57] or lead to a decrease in OXPHOS that can cause abnormal human embryo development [58]. Altered mitochondrial activity and dynamics in embryos have a remarkable capacity to establish enduring alterations to the epigenetic landscape [59]. The mitochondrial count in the oocyte must therefore be optimal and healthy enough to be divided between the different blastomeres, allowing them to have their own mitochondria for the sufficient production of ATP and metabolites essential to their functioning and development until mitochondrial replication starts again [23,43,59,60]. Thus, we speculated that autologous mitochondrial supplementation might be an excellent approach to improving the quality of vitrified-w oocytes and their post-fertilization development.

In regenerative medicine, adipose stem cells are one of the most in-demand cell types because of their capacity to restore mitochondrial function [61,62]. Moreover, adipose stem cells can transfer mitochondria in vitro and in vivo to exhausted cells to reduce ROS levels and increase the number of intact mitochondria [61,63]. Unlike most other cells, oocytes contain a higher number of mitochondria, showing few cristae spherical shapes [64,65]. Interestingly, ASC mitochondria have also demonstrated similar morphological phenotypes to oocytes, including spherical shape and limited cristae [29] (Figure 3D,E), and they are potentially active ones (Figure 3A–C). Therefore, we have selected adipose stem cell mitochondria as the most suitable source of mitochondria for supplementing cryopreserved oocytes in our experiments.

A previous study reported that ASC mitochondria supplementation in aged oocytes could promote oocyte quality, embryo development, and fertility in aged mice, supporting the possibility of using autologous ASC mitochondria to restore the quality of oocytes [29]. Intriguingly, in our study, the ASC mitochondria-supplemented cryopreserved oocytes showed significantly higher embryo development potential than the buffer-injected cont- ICSI group oocytes (Table 1, Figure 4A,D,E). And following the transplantation of 2-cell embryos of both groups into a single recipient, we obtained four pups from the ASC mitochondria supplemented group and one from the cont-ICSI group (data not shown), further confirming successful embryo development. The average range of mitochondrial DNA copy numbers in oocytes depends on animal species, age, and physiological condition, and 161,000+/−73,000 mitochondrial DNA copies are available in the mouse mature oocyte [22,66]. The threshold quantity and the quality of mitochondria are vital for successful fertilization and subsequent embryo formation [67]. Thus, we suggest that mitochondria supplementation may restore the developmental capacity of cryopreserved oocytes through the fulfillment of quantitative mitochondrial demand in early embryos.

The availability of active mitochondria in 2-cell embryos is critical in developing early preimplantation embryos, and defective mitochondria in mature oocytes may prevent their efficacious development [23]. Additionally, reduced ATP content in early embryos may be related to arrested division, abnormal embryonic development, and impaired offspring phenotype [68,69]. In our study, we analyzed intracellular ROS levels and ATP production in 2-cell embryos. Interestingly, the 2-cell embryos of the mito-ICSI group showed significantly higher ATP production ability compared to the cont-ICSI group (Figure 5A), which may have positively influenced blastocyst development potentials. In contrast, the intracellular ROS was comparable in 2-cell embryos of both groups (Figure 5B). Oscillating ROS levels in early embryos is essential for cell cycle regulation. Inhibiting mitochondrial ROS production in early embryos results in cell-cycle arrest [44]. Thus, ROS levels may be highly variable across cell division stages, and it may be challenging to make any assumptions about the quality of early embryos using the intracellular ROS level of randomly selected 2-cell embryos. These results suggest that mitochondria supplementation may have improved early embryo quality and developmental potential by restoring the required optimal energy metabolism, which deteriorated in conventional early embryos developed from cryopreserved oocytes.

We acknowledge that the limitations of this study include a lack of compared development results with fresh oocytes’ ICSI, information on the detailed cellular molecular mechanism behind this improvement of embryo development, and information on transgenerational safety in offspring following mitochondria supplementation. The ICSI-mediated cellular stress may be similar to either fresh or cryopreserved oocytes, and positive influence may be detected from mitochondria supplementation despite the oocyte status. In our future studies, we plan to confirm the detailed cellular mechanism and the transgenerational safety of this procedure. And another limitation of this study is that the average survival rate of oocytes following both cont-ICSI and Mito-ICSI is a little lower than the conventional methods, which might be due to the insertion of an extra buffer with or without mitochondria.

## 5. Conclusions

Oocyte mitochondria are critical cellular organisms that regulate the potentiality of embryo development. In vitro manipulations such as cryopreservation may cause notable oxidative stress and damage the mitochondria, resulting in compromised preimplantation embryo development. The supplementation of adipose stem cell mitochondria can successfully remediate the declined embryo development and live birth potentials caused by cryopreservation-mediated cellular stresses and damages. Thus, we propose that adipose stem cell mitochondria may be effective as an autologous mitochondria source to improve embryo development potential, at least in cryopreserved mouse oocytes.

## Figures and Tables

**Table 1:** Adipose stem cell mitochondria injection improves the blastulation rate of cryopreserved oocytes.

	Cont-ICSI	Mito-ICSI	*p*-Value
Total number of oocytes injected	105	104	
The survival rate after ICSI (%)	50.4 (53/105)	58.6 (61/104)	0.46
Fertilization rate per survived oocytes (%)	75.4 (40/53)	86.8 (53/61)	0.36
2 cell rate per survived oocytes (%)	59 (34/53)	64.3 (44/61)	0.71
Blastulation rate per survived oocytes (%)	24.5 (13/53)	40.9 (25/61)	0.008
Blastulation rate per 2 cell embryos (%)	38.2 (13/34)	56.8 (25/44)	0.02

## Data Availability

The data presented in this study are available on request from the corresponding author.

## Supplementary Materials

The following supporting information can be downloaded at: https://www.mdpi.com/article/10.3390/biom12071008/s1, Figure S1: FACS plots for fresh adipose stem cell isolation and fresh ovarian stem cell isolation.
