# Supplementary material for: Mitochondria Transfer from Adipose Stem Cells Improves the Developmental Potential of Cryopreserved Oocytes
Source: Biomolecules. 2022 Jul 21;12(7):1008. doi: 10.3390/biom12071008 (PMC9313289; doi:10.3390/biom12071008)
Supplement: Supplementary file 1 [file biomolecules-12-01008-s001.zip › biomolecules-1802867-supplementary.pdf]

Article

Mitochondria transfer from adipose stem cells improves the developmental potential of cryopreserved oocytes

Sanath Udayanga Kankanam Gamage[1]*, Shu Hashimoto[2], Yuki Miyamoto[1], Tatsuya Nakano[3], Masaya Yamanaka[3], Akiko Koike[1], Manabu Sato[3], Yoshiharu Morimoto[1]*

1. HORAC Grand Front Osaka Clinic, Osaka, Japan
2. Reproductive Science Institute, Graduate School of Medicine, Osaka Metropolitan University, Osaka, Japan
3. IVF Namba Clinic, Osaka, Japan

Supplementary figure

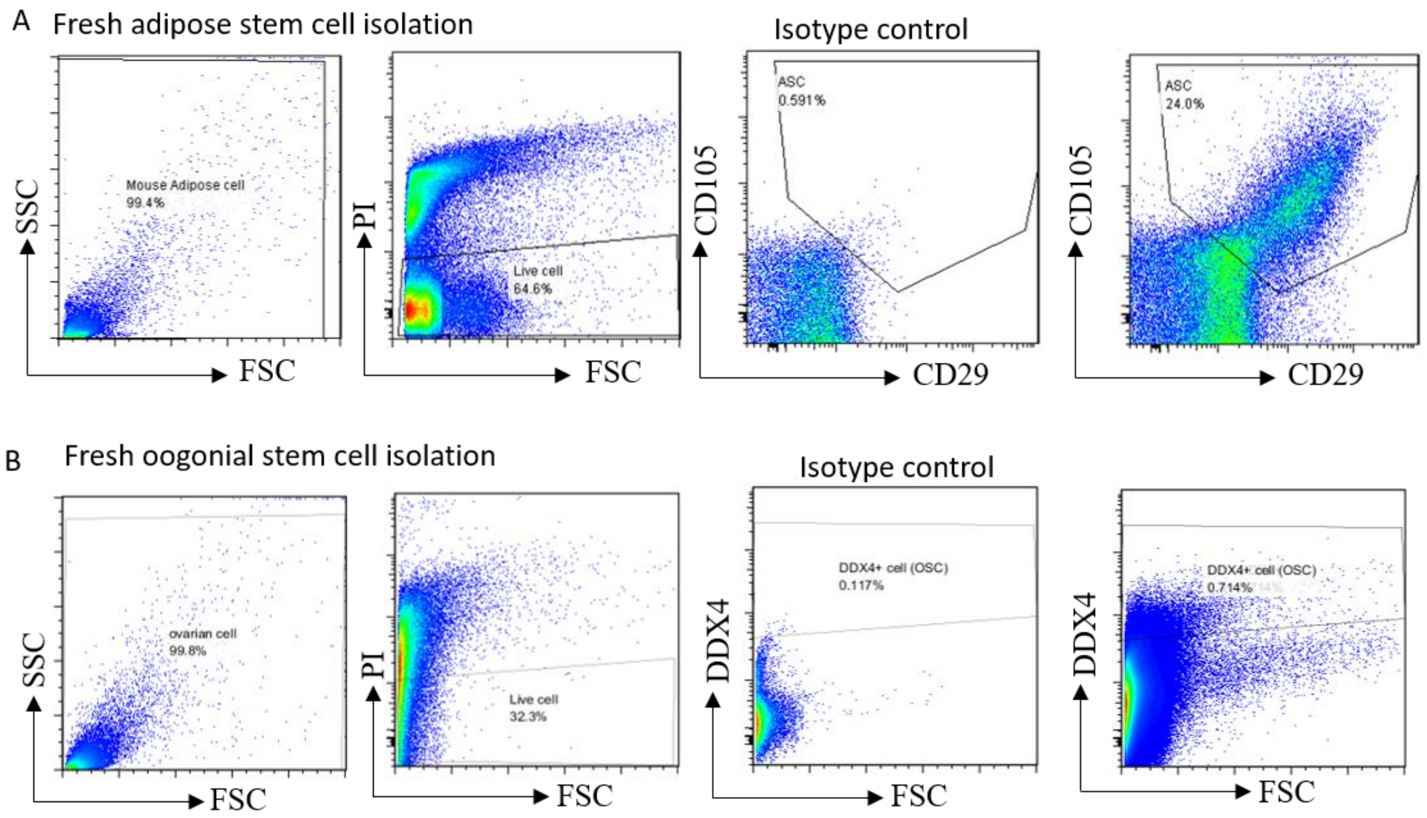

Supplementary Figure S1. FACS plots for fresh adipose stem cell isolation and fresh oogonial stem cell isolation. A. Fresh adipose stem cells were sorted by using FACS. The adipose cell was stained with isotype antibodies or target antibodies (CD105 & CD29) and propidium iodide (PI). ASC population was gated according to the isotype control staining in the PI negative population. **B.** Fresh oogonial stem cells (OSC) were sorted by using DDX4 antibody and isotype control antibody. OSC population was gated according to isotype control staining in PI negative cells.
